# A dataset of chromosomal instability gene signature scores in normal and cancer cells from the human breast

**DOI:** 10.1016/j.dib.2023.109647

**Published:** 2023-10-08

**Authors:** Shahnawaz A. Baba, Shreyas Labhsetwar, Richard Klemke, Jay S. Desgrosellier

**Affiliations:** Department of Pathology, University of California, San Diego, 9500 Gilman Drive, La Jolla, CA 92093, USA

**Keywords:** Cancer stem cell, Chromosomal missegregation, Signaling state, Stress tolerance, Breast cancer subtype, Tumor initiation

## Abstract

These data show the relative amount of chromosomal instability (CIN) in a diverse array of human breast cell types, including non-transformed mammary epithelial cells as well as cancer cell lines. Additional data is also provided from human embryonic and mesenchymal stem cells. To produce this dataset, we compared a published chromosomal instability gene signature against publicly available datasets containing gene expression information for each cell. We then analyzed these data with the Python GSEAPY software package to provide a CIN enrichment score. These data are useful for comparing the relative amounts of CIN in different breast cell types. This includes cells representing the major clinical (ER/PR^+^, HER2^+^ & Triple-negative) as well as intrinsic breast cancer subtypes (Luminal B, HER2+, Basal-like and Claudin-low). Our dataset has a great potential for re-use given the recent surge in interest surrounding the role of CIN in breast cancer. The large size of the dataset, coupled with the diversity of the cell types represented, provides numerous possibilities for future comparisons.

Specifications Table

**Table:** 

Subject	Cancer Research
Specific subject area	Our work focuses on breast cancer stem cells and their role in tumor progression and metastasis.
Data format	Raw
Type of data	Excel spreadsheets
Data collection	Data were collected after comparing each cell type present in the gene expression data files downloaded from NCBI with the published CIN gene signature. The provided CIN enrichment score for each cell type is listed in the Excel spreadsheets deposited. Data was normalized using the trimmed mean of M-values (TMM).
Data source location	• *Institution:* University of California, San Diego *• City/Town/Region:* La Jolla, CA *• Country:* USA *• Latitude and longitude:* 32.876328, −117.236067
Data accessibility	Repository name: UC San Diego Library Digital Collections Data identification number: doi:10.6075/J0R78FDG Direct URL to data: https://library.ucsd.edu/dc/object/bb12054874
Related research article	S.A. Baba, Q. Sun, S. Mugisha, S. Labhsetwar, R. Klemke, J.S. Desgrosellier, Breast cancer stem cells tolerate chromosomal instability during tumor progression via c-Jun/AXL stress signaling, *Heliyon*. In Press.

## 1 Value of the Data

• While CIN is a defining hallmark of cancer, little is known about its relationship with stemness. High levels of CIN are associated with breast cancer evolution and metastasis [1] suggesting that it may be a unique feature of aggressive cells. Tumor-initiating cancer stem cells (CSCs) bearing similarities to adult mammary stem cells are a highly aggressive tumor cell subset that contribute to metastasis and disease progression [2], [3], [4], [5], [6], [7]. This suggests that CSCs may contain high levels of CIN, enhancing their aggressive properties. Thus, we generated this dataset to better understand how CIN levels compare in stem and non-stem cell types.
• These data add value to our original research article by allowing us to assess potential associations between CIN levels and stem-like breast cancer cells and draw important conclusions regarding the impact of CIN on tumor initiation.
• The CIN enrichment scores provided in this dataset are useful for comparing the relative amounts of CIN present in different non-transformed and cancer cell lines from the breast.
• This dataset may be useful to anyone associated with breast cancer research. CIN is a hallmark of cancer and is well described to play a role in tumor evolution, especially in regards to therapeutic resistance [8]. Recent high-profile work has further characterized CIN as a driver of antiviral innate immune signaling in breast cancer cells [9], resulting in tumor progression and metastasis [1]. Thus, CIN is an important area of research in the breast cancer field and this dataset may aid future studies of this topic.
• These data can be used/reused for further insights into associations between CIN levels and numerous additional variables, including cell-of-origin, mutational status, subtype, gene expression, etc.

## 2 Data Description

The deposited dataset consists of two distinct Excel spreadsheets [10]. Each spreadsheet lists the cell names or identifiers across the top of each column and the corresponding enrichment scores (ES) and normalized enrichment scores (NES) underneath. To obtain the scores, we compared a published CIN gene signature [1] to the following gene expression datasets downloaded from NCBI:

Breast cancer cell lines [11]:

https://www.ncbi.nlm.nih.gov/geo/query/acc.cgi?acc=GSE50470

HCC38 sorted cells [12]:

https://www.ncbi.nlm.nih.gov/sra/?term=PRJNA750073

We compared these datasets to the CIN signature by first converting FASTQ files to gene-expression matrices and processing so that only the data were retained. Scores were obtained for each cell type by performing single sample gene set enrichment analysis (GSEA) with Python GSEAPY Library software, and the resulting data were processed into Excel format.

The resulting spreadsheets are labelled and described as follows:

Breast cell lines – Excel spreadsheet listing the CIN enrichment scores for an assortment of breast cancer cell lines as well as non-transformed human cells. Cell names are listed across the top of each column with the corresponding ES and NES scores underneath.

HCC38 sorted breast cancer cells – Excel spreadsheet containing the CIN enrichment scores for HCC38 breast cancer cells sorted according to their cell surface EpCAM (Ep) and integrin αvβ3 (b3) status from three independent experiments. Sorted cells from each experiment were divided into four categories: EpCAM low (lo) versus high (hi) and αvβ3 positive (pos) versus negative (neg). Each cell type belonging to a particular experiment was given a unique Run number (SRR) listed across the top of each column. For quick reference, the sorted cell type is included underneath, followed by the ES and NES scores.

## 3 Experimental Design, Materials and Methods

Publicly available RNA sequencing data from breast cancer and normal mammary cell lines was obtained from NCBI GEO (GSE50470), while data from sorted HCC38 cell populations was downloaded from the NCBI Sequence Read Archive (PRJNA750073). FASTQs were converted to gene-expression matrices and the files were processed to remove all the header information and only retain the data. The CIN gene signature was acquired from Bakhoum et al. [1] and CIN scores were obtained by examining enrichment for the CIN associated gene signature in each cell type represented in the sequencing datasets according to Barbie et al. [13].

To generate the CIN scores for each cell type, we analyzed data with the Python GSEAPY Library (https://gseapy.readthedocs.io/en/latest/). First, input files were read using Python's Pandas library and joined with each other using the ID & amp columns before deleting any unnecessary columns. Ensemble Gene IDs were mapped to their HGNC Symbols using Python's BioMart API (https://pypi.org/project/biomart). Any Ensemble ID which did not have a corresponding HGNC Symbol was dropped. Once we obtained the data frame having HGNC Symbols as rows, samples as columns, and their feature counts as values in all rows, this data frame, along with the CIN gene set was passed to the Single Sample GSEA Python library. The final data comprised 36,866 rows and 106 columns before feeding it into GSEAPY. To determine the enrichment scores (ES), we applied Single Sample GSEA to the final data frame. The experiment was repeated with a normalized version of the data frame, but the normalized enrichment scores (NES) were identical to the ES. GSEAPY output was then processed into Excel format and saved as final results files.

## Limitations

None.

## Ethics statement

We confirm that the authors have read and follow the ethical requirements for publication in Data in Brief and that the current work does not involve human subjects, animal experiments, or any data collected from social media platforms.

## CRediT authorship contribution statement

**Shahnawaz A. Baba:** Conceptualization, Methodology, Writing – original draft. **Shreyas Labhsetwar:** Methodology, Formal analysis, Software. **Richard Klemke:** Methodology, Formal analysis, Software. **Jay S. Desgrosellier:** Conceptualization, Methodology, Formal analysis, Visualization, Investigation, Supervision, Funding acquisition.

## Data Availability

A dataset of chromosomal instability gene signature scores in normal and cancer cells from the human breast (Original data) (UC San Diego Library Digital Collections)
